# Machine Learning Big Data Analysis of the Impact of Air Pollutants on Rhinitis-Related Hospital Visits

**DOI:** 10.3390/toxics11080719

**Published:** 2023-08-21

**Authors:** Soyeon Lee, Changwan Hyun, Minhyeok Lee

**Affiliations:** 1 School of Electrical and Electronics Engineering, Chung-Ang University, Seoul 06974, Republic of Korea; soyeon1608@cau.ac.kr; 2 Department of Urology, Korea University College of Medicine, Seoul 02841, Republic of Korea; gusckddhks@kumc.or.kr

**Keywords:** rhinitis, air pollution, machine learning, hospital visits, carbon monoxide, nitrogen dioxide, ozone, particulate matter, time lag effect, respiratory health

## Abstract

This study seeks to elucidate the intricate relationship between various air pollutants and the incidence of rhinitis in Seoul, South Korea, wherein it leveraged a vast repository of data and machine learning techniques. The dataset comprised more than 93 million hospital visits (*n* = 93,530,064) by rhinitis patients between 2013 and 2017. Daily atmospheric measurements were captured for six major pollutants: PM${}_{10}$, PM${}_{2.5}$, O_3_, NO_2_, CO, and SO_2_. We employed traditional correlation analyses alongside machine learning models, including the least absolute shrinkage and selection operator (LASSO), random forest (RF), and gradient boosting machine (GBM), to dissect the effects of these pollutants and the potential time lag in their symptom manifestation. Our analyses revealed that CO showed the strongest positive correlation with hospital visits across all three categories, with a notable significance in the 4-day lag analysis. NO_2_ also exhibited a substantial positive association, particularly with outpatient visits and hospital admissions and especially in the 4-day lag analysis. Interestingly, O_3_ demonstrated mixed results. Both PM${}_{10}$ and PM${}_{2.5}$ showed significant correlations with the different types of hospital visits, thus underlining their potential to exacerbate rhinitis symptoms. This study thus underscores the deleterious impacts of air pollution on respiratory health, thereby highlighting the importance of reducing pollutant levels and developing strategies to minimize rhinitis-related hospital visits. Further research considering other environmental factors and individual patient characteristics will enhance our understanding of these intricate dynamics.

## 1. Introduction

Within the intricate tapestry of environmental and health sciences, the interplay of various external and internal factors is paramount [1,2,3]. A case in point is the condition of rhinitis, which is a prevalent yet often overlooked disorder that acts as a crucial intersection for this interdisciplinary exploration. Rhinitis, which is manifested by clinical features such as nasal congestion, sneezing, and sinus pressure, holds substantial global prevalence, wherein it impacts a substantial portion of the human populace [4,5,6]. Its complex etiology, from allergenic to irritant triggers, genetic susceptibility, and environmental pollution, merits concentrated scrutiny.

Air pollution, which is a pervasive and escalating global issue, has significant ramifications for public health [7,8,9]. Being composed of an array of constituents, including particulate matter, ground-level ozone (O_3_), carbon monoxide (CO), sulfur dioxide (SO_2_), and nitrogen dioxide (NO_2_), it poses a multifarious hazard [10,11,12,13,14,15]. This unseen adversary often goes unnoticed until its deleterious effects become manifest, especially in urban settings where industrialization and urbanization amplify these pollutant concentrations and their associated impacts.

The intricate relationship between rhinitis and air pollution presents a captivating frontier for research. While it is known that rhinitis patients exhibit heightened sensitivity to environmental precipitants [16,17,18], the nuanced contributions of specific types of air pollutants in the exacerbation of rhinitis symptoms has yet to be exhaustively investigated. Furthermore, the temporal association between the exposure to pollutants and the onset or amplification of symptoms, colloquially known as the time-lag effect, remains a largely uncharted domain.

Our research aims to mitigate these knowledge deficiencies by leveraging the capabilities of machine learning, which is a methodological approach that has been celebrated for its aptitude in dealing with high-dimensional and intricate data. We seek to delineate the relationships between diverse air pollutants and rhinitis, as well as to unmask the potential time lag effect. We utilized an extensive dataset, comprising daily atmospheric measurements coupled with hospital visits by rhinitis patients, which amounted to a total exceeding 93 million hospital visits due to rhinitis across the span of 2013 to 2017 from Seoul, South Korea.

In recent years, the advent and ascension of machine learning techniques have catalyzed a revolution in the analysis of biomedical big data [19,20,21,22]. The ability to process and derive meaningful insights from large-scale, complex data has paved the way for a more nuanced understanding of disease patterns, genetic underpinnings, and the impacts of environmental factors on health [23,24,25,26]. In the context of our study, machine learning offers a novel approach to understanding the intricate dynamics between air pollution and rhinitis, thus aiding in the extraction of valuable insights from the vast amount of data we have amassed.

By forging a comprehensive comprehension of these associations, we aim to bolster preventive strategies, augment public health guidelines, and ultimately facilitate the improved management and treatment of rhinitis. Consequently, this investigation is not merely an academic endeavor, but is also an integral step towards ameliorating global respiratory health amid the rising tide of environmental challenges.

## 2. Materials and Methods

### 2.1. The Comprehensive Rhinitis Patient Visit Database in Seoul

Located in the heartland of South Korea, Seoul is a thriving metropolis, which is home to around 10 million individuals. The investigation presented in this paper capitalizes on an extensive database that captures hospital visitations by rhinitis patients within this populous city.

In South Korea, national health insurance is not optional but a requirement for every citizen. As a result, the National Health Insurance Service (NHIS) of South Korea finds itself in the unique position of holding comprehensive medical records for every individual in the nation. In addition, South Korea boasts of a robust healthcare system that is characterized by top-tier accessibility. This conducive environment frequently prompts patients with even mild rhinitis to seek medical attention at hospitals.

To facilitate research similar to that in the current study, the NHIS has meticulously curated a specific database catering to rhinitis patients. This repository incorporates a multitude of variables, such as daily counts of outpatient visits, the number of hospital admissions, and emergency department visits.

Examining the particulars of the data available within the timeframe of five years, spanning from 2013 to 2017, the incidence of hospital visits made by rhinitis patients in Seoul reached an astonishing tally of nearly 112 million cases. For the purpose of our analysis, we made the decision to exclude weekend data, as the observed patterns markedly deviated from those during weekdays, which could primarily be attributed to the routine shutdown of numerous hospitals over the weekend. Following similar reasoning, we also omitted data corresponding to the 63 public holidays observed in South Korea between 2013 and 2017.

**Figure 1:**
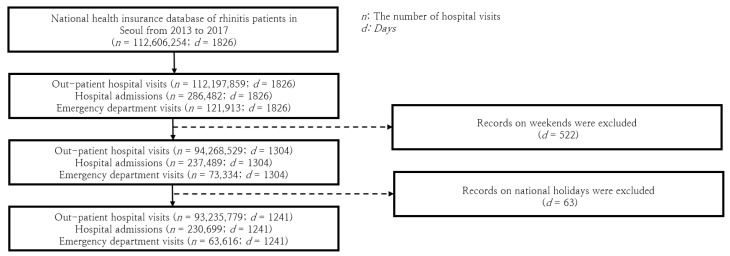
Diagram illustrating the data curation process, from raw hospital visitation data to the final dataset utilized in the analysis, after excluding weekends and holidays data.

In the wake of these exclusions, our final dataset for analysis encompassed 93,235,779 outpatient visits, 230,699 hospital admissions, and 63,616 emergency department visits spread across a total of 1241 days. To prepare this massive dataset for statistical processing, we applied a log normalization technique to convert the originally skewed data distributions into a more tractable Gaussian distribution. The entirety of the database curation process is encapsulated visually in Figure 1, thus offering an overview for reference.

### 2.2. Database of Daily Atmospheric Environmental Details

The backbone of our research is a comprehensive database that documents daily environmental atmospheric variables from 2013 to 2017 across 25 distinct locales within Seoul. The database captures daily average values for key air pollutants, namely, PM${}_{10}$, PM${}_{2.5}$, O_3_, NO_2_, CO, and SO_2_, at each of the specified locations. For congruity with the hospital visit database, we excluded data corresponding to weekends and national holidays.

### 2.3. Analytical Approach: Combining Traditional Statistics and Machine Learning

To investigate the relationships inherent in our data, we deployed a multifaceted analytical approach. Firstly, correlation analyses were performed using Pearson and Spearman correlation coefficients. Alongside this traditional statistical approach, we employed machine learning techniques, specifically the least absolute shrinkage and selection operator (LASSO) [27], random forest (RF) [28] and gradient boosting machine (GBM) [29], to analyze the effects of air pollutants and the time lag in hospital visits by rhinitis patients.

#### 2.3.1. Pearson and Spearman Correlations

In assessing the relationships between our variables, we employed both Pearson and Spearman correlations. The Pearson correlation coefficient measures the linear relationship between two datasets and is defined by the following formula:

(1) $$\rho_{XY}=\frac{Cov(X,Y)}{\sigma_{X}\sigma_{Y}}$$

Here, Cov(X,Y) is the covariance of variables *X* and *Y*, while $\sigma_{X}$ and $\sigma_{Y}$ are their respective standard deviations. The Pearson coefficient ranges between −1 and 1, with 1 signifying a perfect positive linear relationship, −1 indicating a perfect negative linear relationship, and 0 indicating no linear correlation.

The Spearman correlation, on the other hand, measures the monotonic relationship between two datasets, which is not limited to linear relationships. It ranks the data points and uses these ranks to calculate correlation. High values (close to 1) suggest a strong, positive monotonic relationship, low values (close to −1) suggest a strong, negative monotonic relationship, and values close to zero suggest a weak or nonmonotonic relationship.

#### 2.3.2. Least Absolute Shrinkage and Selection Operator (LASSO)

LASSO is a regularization technique that induces model parsimony by shrinking certain regression coefficients towards zero, thereby effectively performing feature selection. It works by adding a penalty that is equivalent to the absolute value of the magnitude of coefficients to the loss function, as is illustrated by the following formula:

(2) $$\underset{\beta }{\mathrm{argmin}}\left\{\sum_{i=1}^{n}{\left(y_{i}-\sum_{j}x_{ij}\beta_{j}\right)}^{2}\right\}\;\mathrm{subject}\;\mathrm{to}\;\sum_{j=1}^{p}\left|\beta_{j}\right|\leq s$$

In the above equation, β is the coefficient vector, and *s* is a predefined parameter controlling the level of regularization. Large absolute values of β signify the contribution of the corresponding variable to the prediction. Conversely, as *s* grows larger, estimates of β shrink towards zero, thereby signifying less contribution from the associated variable.

#### 2.3.3. Random Forest (RF)

Random forest is a tree-based ensemble model that can be used for both classification and regression tasks. It operates by generating a multitude of decision trees, with each branching being based on a given criterion until a termination condition is met. A key feature of the RF model is its ability to provide a measure of the feature importance, thus quantifying the contribution of each variable to the model.

The importance of the *i*th feature ($I(f_{i})$) is calculated as follows:

(3) $$I\left(f_{i}\right)=\frac{\sum_{j}w_{j}\cdot G\left(C_{j}\right)-w_{j_{left}}\cdot G\left(C_{j_{left}}\right)-w_{j_{right}}\cdot G\left(C_{j_{right}}\right)}{\sum_{k}I\left(C_{k}\right)}$$

Here, $C_{j}$ refers to the importance of node $C_{j}$, while *w* is the weight of node $C_{j}$, which is represented as the ratio of the number of samples at node $C_{j}$ to the total sample size. The denominator $\sum_{k}I\left(C_{k}\right)$ is the total importance of all nodes. The importance $I(f_{i})$ in RF corresponds to the average of all $I(f_{i})$ values across each individual tree, thus offering a measure of variable importance.

#### 2.3.4. Gradient Boosting Machine (GBM)

The gradient boosting machine, or GBM, is a powerful ensemble machine learning algorithm that constructs models in a stage-wise fashion, thus optimizing an arbitrary differentiable loss function. Similar to RF, GBM can provide a measure of feature importance.

The importance of each variable in GBM is determined by the number of times a variable is selected for splitting, which is weighted by the squared improvement to the model as a result of each split and averaged over all trees. High values of feature importance imply a more significant role of the corresponding feature in the model, whereas low values suggest a lesser contribution.

#### 2.3.5. Interpreting Coefficients and Importance Measures

While interpreting the values in the models, it is critical to remember that these values do not imply causation, but merely association. Regarding LASSO, high absolute coefficient values indicate features that significantly contribute to predicting the target variable. However, coefficients shrunk to zero are not necessarily irrelevant to the prediction. Their exclusion from the LASSO model only implies that their contribution is not significant when considering the penalty term.

In the case of RF and GBM, high feature importance signifies that a variable significantly contributes to the prediction of the target variable across the trees in the forest or the iterations of boosting. Conversely, a low importance measure suggests that the feature does not significantly contribute to the prediction in the context of the other features. For all these models, the target variable of our machine learning models is the number of rhinitis patients, with air pollutants serving as inputs.

## 3. Results

### 3.1. Exploratory Data Analysis

#### 3.1.1. Air Pollutants Correlations

**Figure 2:**
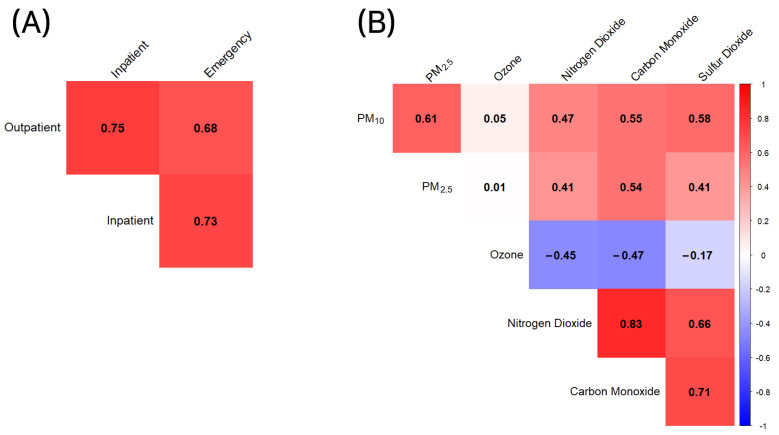
Correlation matrix showcasing relationships between the hospital visit types and relationships among different air pollutants measured in the study. (**A**) Correlation matrix between the hospital visit types. (**B**) Correlation matrix among different air pollutants.

Our initial exploration of the data began by investigating the correlation matrix of the various types of pollutants. The correlation matrix, which is shown in Figure 2, clearly illustrates the relationships among different pollutants.

Specifically, the Pearson correlation between PM${}_{10}$ and PM${}_{2.5}$ was found to be 0.6119, thus indicating a moderate positive correlation. Both pollutants also demonstrated a positive correlation with NO_2_, carbon monoxide, and SO_2_, albeit to a varying degree.

Interestingly, the correlation between ozone and other pollutants mostly trended in the opposite direction. The ozone displayed a weak positive correlation with PM${}_{10}$ and PM${}_{2.5}$ and had a moderate negative correlation with NO_2_, CO, and SO_2_.

#### 3.1.2. Hospital Visit Correlations

We next examined the correlation matrix between different types of hospital visits, including outpatient visits, inpatient admissions, and emergency department visits. As expected, a relatively strong positive correlation was found between the different types of visits, thereby suggesting that days with high outpatient visits also tended to have high inpatient admissions and emergency department visits.

#### 3.1.3. Pollutants and Patient Visits

**Figure 3:**
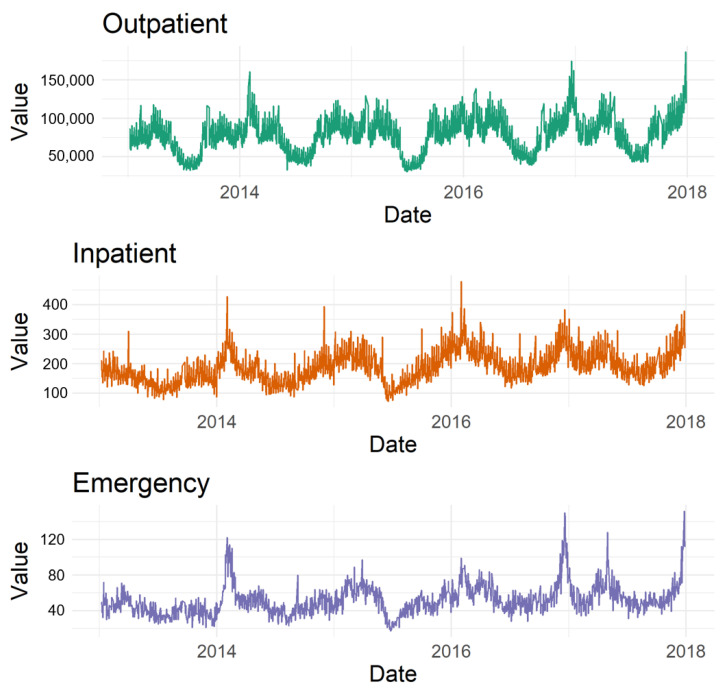
Graphical representation of the daily patient visits to outpatient, inpatient, and emergency departments over the study period.

**Figure 4:**
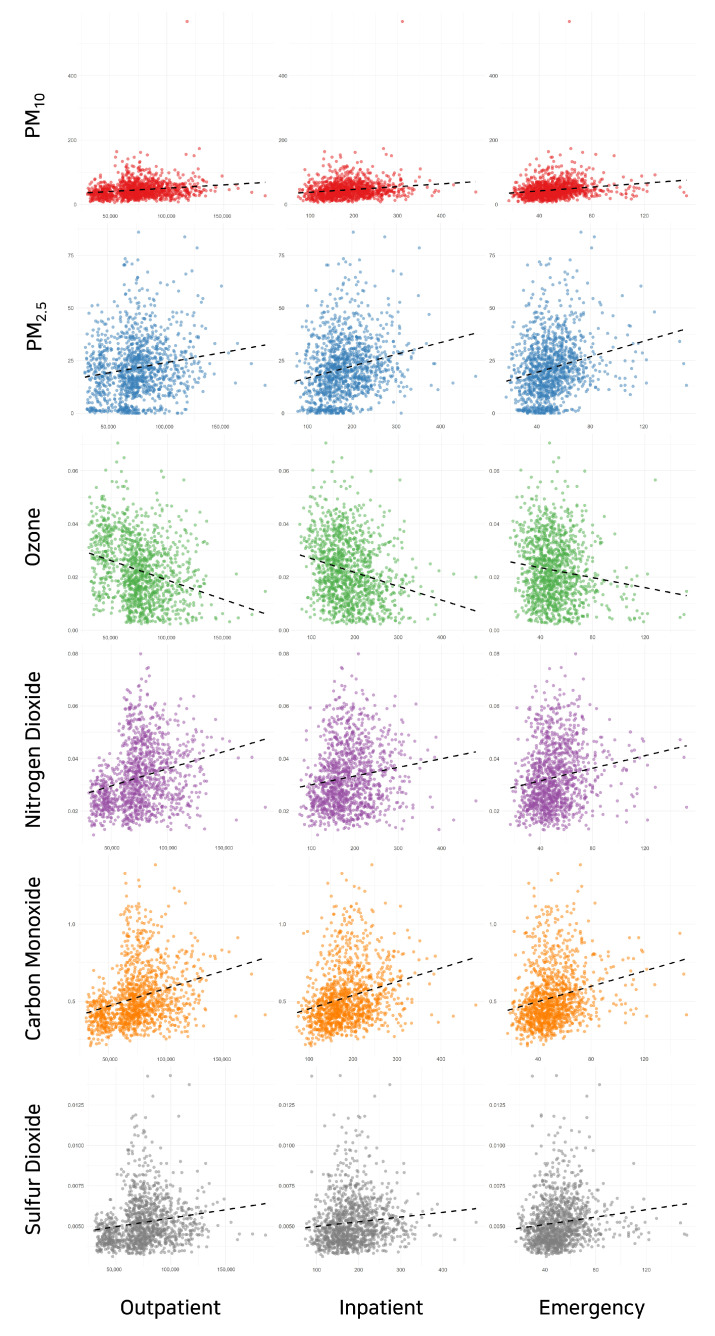
Scatter plots depicting the relationships between each type of pollutant and each type of hospital visit (outpatient visits, inpatient admissions, and emergency department visits).

We calculated the average daily patient visits for the outpatient, inpatient, and emergency departments. Our results showed an average of around 75,000 outpatient visits, 180 inpatient admissions, and 50 emergency department visits per day. Detailed date-wise trends for these averages are depicted in Figure 3. Finally, we investigated the relationships between each type of pollutant and each type of hospital visit. Scatter plots of these relationships were generated and are shown in Figure 4.

### 3.2. Analysis of Hospital Visits and Air Pollutants Using Statistical Analysis

#### 3.2.1. Pearson Correlation Analysis

**Figure 5:**
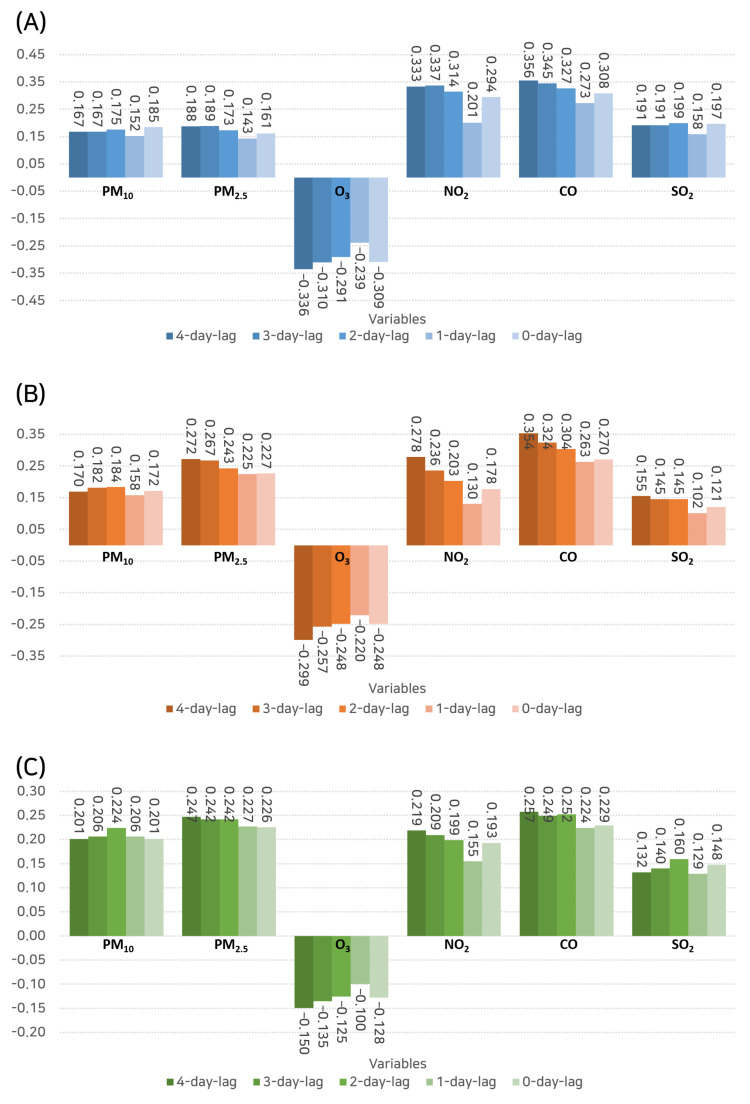
Bar graph representing the Pearson correlation coefficients between the levels of various air pollutants and the number of hospital visits for rhinitis considering time lags from 0 to 4 days. (**A**) Outpatient visits. (**B**) Inpatient admissions. (**C**) Emergency department visits.

The Pearson correlation coefficients, depicted in Figure 5, provide a numerical measure of the linear relationships between the levels of various air pollutants and the number of hospital visits for rhinitis, which consider time lags from 0 to 4 days.

For outpatient hospital visits, CO showed the highest correlation coefficient (r = 0.356) at a 4-day lag, thus indicating a significant positive linear relationship. This was followed closely by NO_2_ (r = 0.333), which was also at a 4-day lag. These findings suggest that the impact of these pollutants on outpatient visits might be more pronounced after a few days from exposure.

In terms of hospital admissions, CO again stood out with the highest correlation (r = 0.354) at a 4-day lag. PM${}_{2.5}$ demonstrated the second highest positive correlation (r = 0.272), which was also at a 4-day lag. This could hint towards a possible delay in the manifestation of symptoms that are severe enough to require hospital admission after exposure to these pollutants.

Interestingly, in the context of emergency department visits, PM${}_{2.5}$ (r = 0.247) and CO (r = 0.257), both measured at a 4-day lag, showed the highest correlation coefficients. This finding further emphasizes the impact of these pollutants on severe symptoms that require immediate medical attention.

#### 3.2.2. Spearman Correlation Analysis

**Figure 6:**
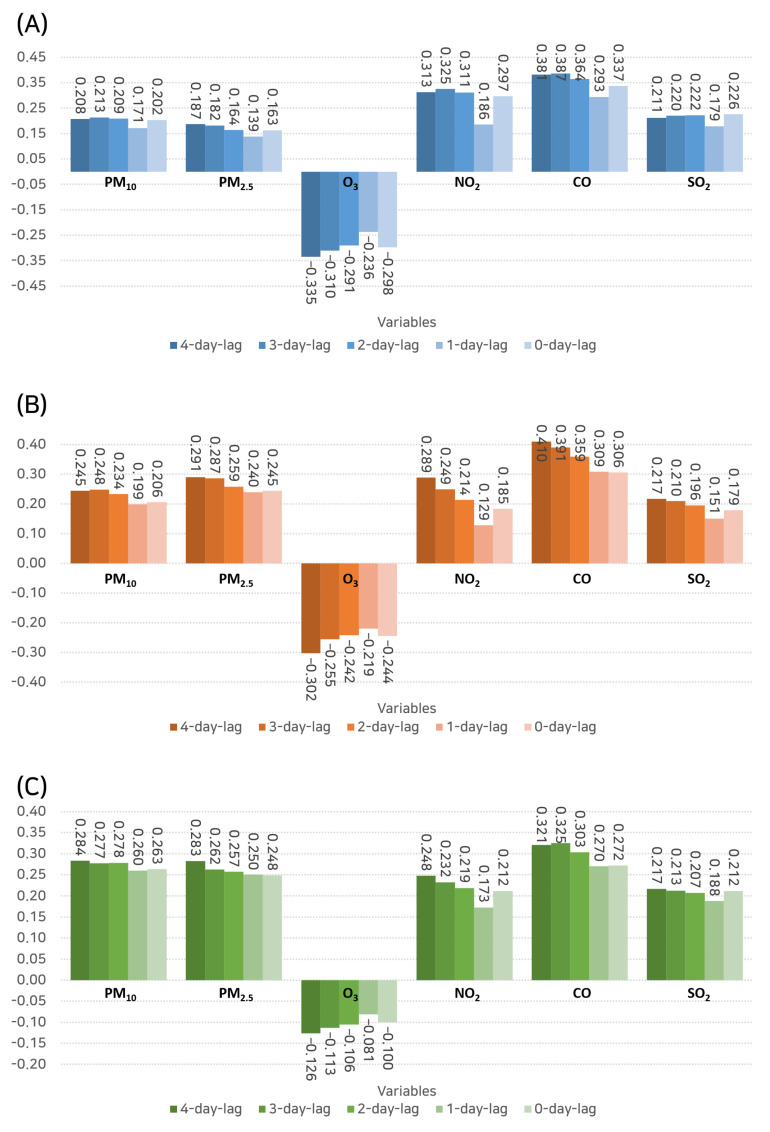
Bar graph illustrating the Spearman correlation coefficients between pollutant levels and the number of hospital visits, thus measuring the monotonic relationships. (**A**) Outpatient visits. (**B**) Inpatient admissions. (**C**) Emergency department visits.

Spearman correlation coefficients, as shown in Figure 6, were also calculated to measure the monotonic relationships between the pollutant levels and the number of hospital visits.

In the case of the outpatient hospital visits, CO once again had the highest correlation (r = 0.381) at a 4-day lag, followed by PM${}_{10}$ (r = 0.208). This reinforces our finding from the Pearson correlation analysis regarding the delayed impact of these pollutants on outpatient visits.

Hospital admissions exhibited the highest Spearman correlation with CO (r = 0.410) at a 4-day lag and PM${}_{10}$ (r = 0.245) at a 4-day lag as well. This observation aligns with the Pearson analysis, thus underscoring the possible delayed impact of these pollutants on severe rhinitis symptoms that necessitate hospital admission.

Lastly, emergency department visits were most strongly correlated with PM${}_{10}$ (r = 0.284) at a 4-day lag and CO (r = 0.321) at the same lag. These findings further emphasize the role of these pollutants in causing severe symptoms requiring emergency care and again emphasize the significant delayed effect.

### 3.3. Analysis of Hospital Visits and Air Pollutants Using Machine Learning Analysis

In the present study, we utilized several machine learning techniques, namely, LASSO, RF, and GBM, to further investigate the effects of air pollutants on rhinitis-related hospital visits.

#### 3.3.1. LASSO Analysis

**Figure 7:**
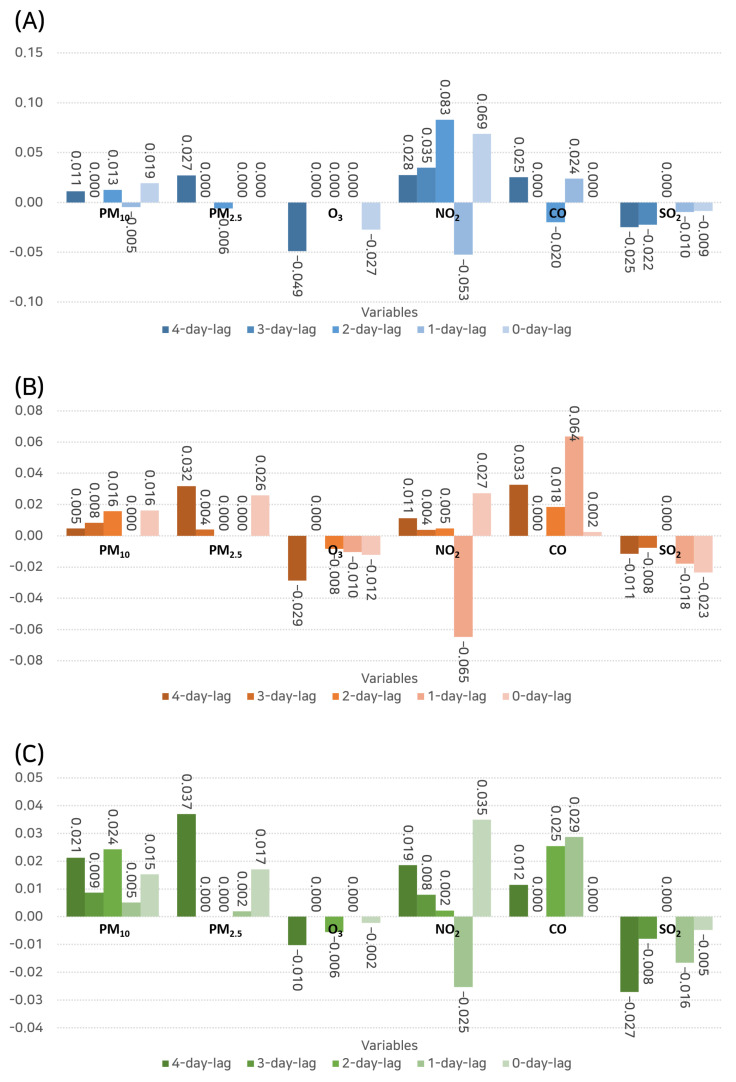
Results of the LASSO regression analysis highlighting the potential relationships between air pollutants and hospital visits at various time lags. (**A**) Outpatient visits. (**B**) Inpatient admissions. (**C**) Emergency department visits.

LASSO regression, which is an advantageous regularization and variable selection method, was deployed to provide a comprehensive and quantitative analysis of the potential relationships (Figure 7). Notably, for outpatient hospital visits, at a 4-day lag, PM${}_{2.5}$ demonstrated the highest positive coefficient (0.027), thus hinting towards a potential link between this pollutant and an increase in outpatient visits. Interestingly, ozone (O_3_) was the only pollutant to show a negative coefficient (−0.049), thereby indicating a possible inverse relationship. At the 1-day lag, NO_2_ and CO showed the most significant positive coefficients (0.024 and 0.028, respectively), thus suggesting that these pollutants might have a more immediate impact on outpatient visits.

In terms of hospital admissions, at a 4-day lag, CO and PM${}_{2.5}$ indicated the highest positive coefficients (0.033 and 0.032, respectively), thus reinforcing the results observed from the correlation analysis. At a 1-day lag, CO showed a remarkably high coefficient of 0.064, which potentially signifies a more immediate role of this pollutant in severe symptom manifestation.

For emergency department visits, the 4-day lag showed the highest positive coefficients for PM${}_{2.5}$ (0.037) and PM${}_{10}$ (0.021). This could reflect the role of these pollutants in exacerbating severe symptoms, thereby necessitating immediate medical intervention.

#### 3.3.2. Random Forest Analysis

**Figure 8:**
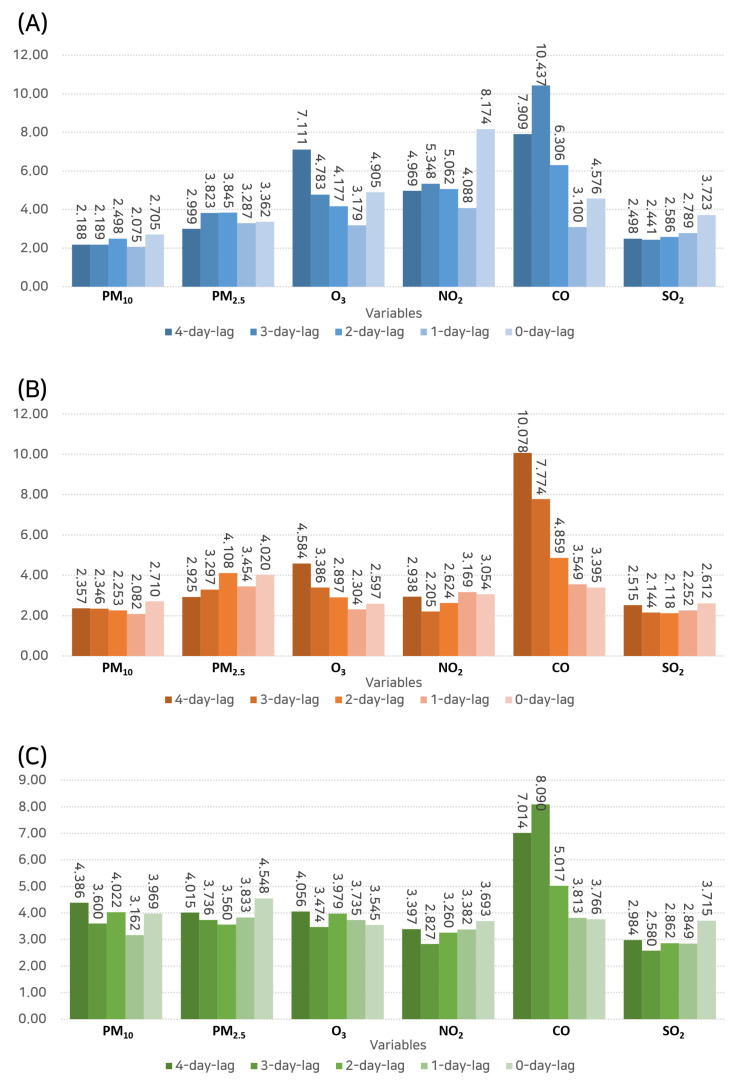
Results of the random forest analysis depicting the feature importance of various pollutants in predicting hospital visits. (**A**) Outpatient visits. (**B**) Inpatient admissions. (**C**) Emergency department visits.

RF, which is a powerful ensemble learning method, was utilized to determine the feature importance of various pollutants in predicting hospital visits (Figure 8). In relation to outpatient visits, CO at a 3-day lag was the most influential variable (importance: 10.437), followed by O_3_ at a 4-day lag (importance: 7.111). This suggests the significant role of these pollutants, specifically CO, in increasing outpatient visits for rhinitis.

When considering hospital admissions, CO showed the highest importance again, but interestingly at a 4-day lag (importance: 10.078), thus indicating a delayed effect of this pollutant. PM${}_{2.5}$ at a 2-day lag was also identified as an important feature (importance: 4.108), thus demonstrating its potential impact on hospital admissions.

For emergency department visits, CO was the most critical feature again at a 3-day lag (importance: 8.090), followed by PM${}_{10}$ at a 4-day lag (importance: 4.386). This underscores the role of these pollutants in causing severe symptoms that require immediate emergency care.

#### 3.3.3. Gradient Boosting Machine Analysis

**Figure 9:**
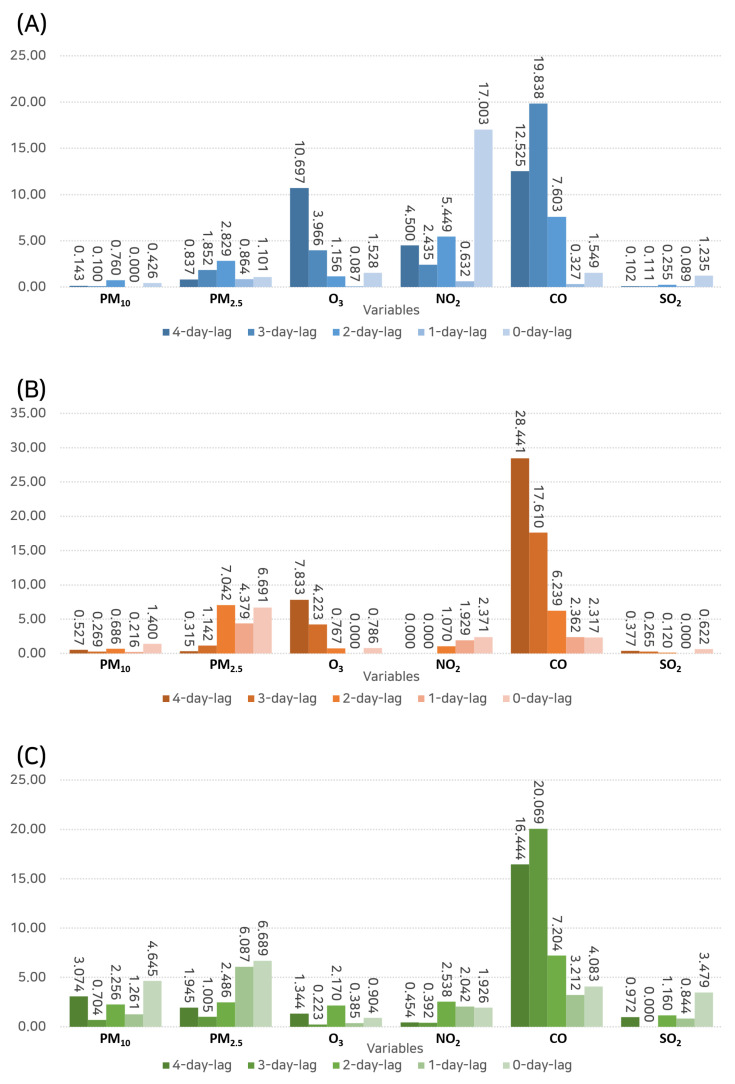
Results of the gradient boosting machine analysis depicting the feature importance of various pollutants in predicting hospital visits. (**A**) Outpatient visits. (**B**) Inpatient admissions. (**C**) Emergency department visits.

The GBM, which is an advanced machine learning algorithm that combines weak prediction models to build a strong predictive model, was used for further analysis (Figure 9).

With regard to outpatient hospital visits, at a 4-day lag, CO demonstrated the highest importance (12.525), followed by O_3_ (10.697). Interestingly, at a 3-day lag, CO exhibited an even higher importance (19.838), thus suggesting its significant and possibly immediate influence on outpatient visits.

When focusing on hospital admissions, CO stood out at a 4-day lag (importance: 28.441), which was substantially higher than other variables, thereby illustrating its possible delay in impacting the severe symptoms that necessitate admission.

In terms of emergency department visits, CO once again exhibited the highest importance at a 4-day lag (importance: 16.444), followed by O_3_ at a 2-day lag (importance: 2.170). This finding further emphasizes the role of these pollutants, especially CO, in causing severe symptoms that require urgent attention.

## 4. Discussion

This investigation utilized a combination of statistical analyses and machine learning algorithms to scrutinize the relationship between various types of air pollutants and the frequency of hospital visits by rhinitis patients. The analysis demonstrated a clear association between elevated levels of certain pollutants and an increase in different types of hospital visits. These findings align with previous studies demonstrating the harmful health impacts of air pollution, especially with respect to respiratory conditions such as rhinitis. This analysis provides a broader understanding of these relationships by considering multiple pollutants simultaneously and incorporating the effect of delayed symptom manifestation following exposure to these pollutants.

Among the pollutants examined, CO consistently emerged as the most significant in terms of its association with hospital visits. This was demonstrated across multiple types of visits, outpatient visits, hospital admissions, and emergency department visits. Notably, CO was especially prominent in the 4-day lag analysis, thereby implying a delayed response to this pollutant. CO, as a common air pollutant, is known to interfere with the oxygen carrying capacity of the blood, thus potentially causing hypoxia, which can exacerbate respiratory symptoms. The findings underscore the importance of continued efforts to monitor and reduce CO levels in the environment to prevent adverse health impacts.

NO_2_ also demonstrated a considerable correlation with outpatient visits and hospital admissions, particularly in the 4-day lag analysis. It is imperative to clarify that NO_2_ is not directly emitted by traffic or burning fossil fuels. Rather, emissions primarily contain NO, which is subsequently oxidized to NO_2_ by the ozone and other peroxides after emissions, which is a process that may exhibit time lags (NO → NO_2_). NO_2_ is known to cause inflammation and damage to the airways, thus potentially worsening rhinitis symptoms. The findings underscore the importance of understanding these chemical dynamics and suggest the necessity to mitigate NO emissions, along with establishing more stringent air quality guidelines to protect individuals, particularly those with existing respiratory conditions.

Contrastingly, O_3_ showed mixed results, with a negative correlation observed in the LASSO analysis for outpatient visits. O_3_ is a primary constituent of smog, and high levels can trigger a variety of health problems, including chest pain, coughing, throat irritation, and airway inflammation [30,31,32]. However, the negative correlation might suggest that other pollutants have a stronger immediate impact on rhinitis symptoms, or there may potentially be mitigating factors related to O_3_ exposure that were not accounted for in this study.

In considering the unexpected negative correlation with O_3_ observed in our study, it is essential to recognize that this particular pollutant is predominantly present during the middle of the day, which is a time typically marked by a decrease in other pollutant concentrations. This daily fluctuation of O_3_ may be perceived as having a potential bearing on the correlation; however, our analysis incorporated the average concentration per day, thereby effectively negating the midday spike’s impact on the results.

It is noteworthy that the middle of the day represents a period of heightened outdoor activity, thus possibly amplifying the effects of O_3_ exposure. In a scenario where O_3_ has a detrimental effect on rhinitis, one might anticipate a distorted correlation between patient visits and the O_3_ concentration, which would be possibly skewed in a higher direction compared to other substances. Nevertheless, our findings revealed the opposite, thereby contributing to the complexity of interpreting the O_3_ relationship.

Upon reflection, it may be posited that ozone’s distinct behavior is modulated by seasonal factors. Ozone is heavily influenced by solar radiation, which exhibits variability across seasons. A potential correlation may exist between the rise in rhinitis during the winter months and the concurrent decrease in solar radiation, thus leading to a reduction in O_3_ concentration. This seasonal modulation of O_3_ may have manifested in our results as a negative correlation.

The aforementioned observations regarding O_3_ call attention to the intricate and multifaceted nature of environmental health interactions. While the negative correlation with O_3_ appears incongruent with conventional understanding, it emphasizes the necessity for a more nuanced examination of pollutant dynamics. The present study’s findings pertaining to O_3_ should be interpreted with caution, thereby recognizing that they introduce new complexities rather than definitive conclusions. Further research that is inclusive of seasonal analyses and possibly considers diurnal variations will be indispensable to unravel the underlying mechanisms governing this perplexing association.

The analysis also revealed a significant role of PM${}_{2.5}$ and PM${}_{10}$ in association with different types of hospital visits. Fine particulate matter, particularly PM${}_{2.5}$, is capable of penetrating deep into the respiratory tract, thereby causing or exacerbating respiratory diseases [33,34,35]. Given their broad sources of emission, ranging from industrial processes to natural phenomena, controlling and monitoring these pollutants present a substantial challenge. However, considering their potential to trigger severe rhinitis symptoms that require immediate medical attention, efforts should be heightened to address this.

A pertinent aspect that merits attention in analyzing the impact of air pollutants on rhinitis-related hospital visits is the potential correlation with traffic density and composition. Traffic emissions are known to be a major source of various pollutants, including PM${}_{2.5}$ and CO, that were central to this study’s findings. Particularly in emerging African countries, there has been evidence of the characterization of ambient aerosols and the assessment of cytotoxicity near high- and low-density traffic sites. A study by Sadiq et al. [36] in Kano State, Nigeria, showed that 51.7% of particles were classified as PM${}_{2.5}$, with significant concentrations at mixed sites comprising both urban and industrial areas. These particulates, which are mainly composed of elements such as Si, Al, Ca, Ce, Ti, Fe, Cl, Pb, and Mn, have been shown to have a direct impact on health, as the proximity to traffic sites led to observed worsening health conditions in the region.

Moreover, traffic density and composition are not only restricted to emerging nations. Even the control measures during the COVID-19 outbreak in China led to reduced traffic, thus resulting in significant decreases in the concentrations of pollutants such as NO_2_, SO_2_, and CO [37]. These reductions were particularly pronounced in highly populated areas with intensive anthropogenic activities. Ground-based observations also supported these findings, thereby demonstrating a significant decrease in the concentrations of NO_2_, SO_2_, CO, PM${}_{2.5}$, and PM${}_{10}$ during the containment period. However, the effect varied across different regions, thus emphasizing the importance of considering the spatial variations in traffic density and composition.

In the context of our study, these insights imply that traffic density and composition can be vital contributors to the observed associations between pollutants and rhinitis-related hospital visits. They also underline the importance of urban planning and emissions control to manage the levels of these pollutants. The spatial distribution of traffic and the corresponding emission characteristics may provide a deeper layer of complexity to the intricate relationship between air quality and respiratory health. Thus, integrating traffic-related data into future analyses may present a more nuanced understanding of the underlying mechanisms driving the observed correlations. This could lead to more effective strategies for reducing pollutant levels and to ultimately minimizing rhinitis-related hospital visits, thereby considering the broader socioeconomic and infrastructural aspects of urban development and transportation.

The observed trends of the time lags between pollutant exposure and hospital visits suggest that the impact of these pollutants may not be immediate. This could be attributed to the delayed inflammatory response to pollutant exposure or the progressive nature of symptom aggravation that eventually leads to the necessity of medical care. This highlights the importance of considering such lags in future research and potentially in the development of warning systems for individuals with respiratory conditions.

However, this study is not without limitations. The analysis was reliant on hospital visit data, which might not fully reflect the severity of the symptoms experienced by patients. Additionally, while a range of pollutants was included in the analysis, other environmental factors such as temperature and humidity were not considered. These factors could potentially influence both the levels of pollutants and the frequency of rhinitis symptoms. Further research could expand on this study by considering these factors and conducting subgroup analyses based on demographic and clinical characteristics to provide a more nuanced understanding of these relationships.

Additionally, the scope of this study could be broadened by employing a more extensive range of machine learning algorithms, thereby potentially enhancing the confidence in the accuracy of the findings. The utilization of a maximum amount of ML algorithms has been proven effective in similar contexts, such as the study conducted by Mirri et al. [38], where a broad array of ML algorithms was employed to investigate the potential correlation between particulate pollution and the spread of COVID-19 in Emilia-Romagna, Italy. This approach achieved a promising 90% accuracy value in predicting the virus’s possible resurgence based on the presence of particulate pollutants such as PM${}_{2.5}$, PM${}_{10}$, and NO_2_. Considering the similar biochemical components examined in our study, the implementation of a more diverse set of ML algorithms, as outlined in the aforementioned work, may further strengthen our understanding of the impact of air pollutants on rhinitis-related hospital visits. This warrants consideration in future research endeavors and underscores the potential to expand the experimental design to attain a more comprehensive and robust analysis.

## 5. Conclusions

This study has made substantial strides in illuminating the complex dynamics between various types of air pollutants and the frequency of hospital visits by rhinitis patients. Our findings have revealed several noteworthy associations. Specifically, elevated levels of CO and NO_2_ were consistently linked with an increase in outpatient visits, hospital admissions, and emergency department visits. The associations were particularly prominent in the 4-day lag analysis, thereby suggesting a time lag effect in the symptom manifestation following exposure to these pollutants.

Particulate matters, PM${}_{2.5}$ and PM${}_{10}$, also presented a significant correlation with the frequency of hospital visits. Given their ability to penetrate deep into the respiratory tract and aggravate respiratory symptoms, this finding underscores the need for meticulous monitoring and stringent control measures to limit their emission. In contrast, the role of O_3_ was found to be more nuanced, which showed a negative correlation in the LASSO analysis for outpatient visits. Further research could help to shed light on the factors underlying this observation.

While our study has provided valuable insights into the relationships between air pollutants and rhinitis-related hospital visits, it is important to note that several environmental factors such as temperature and humidity were not considered in our analysis. Future studies could aim to incorporate these variables to gain a more holistic understanding of the phenomena.

## Data Availability

The national health insurance data and atmospheric environmental data used in this study are public data that are available at https://nhiss.nhis.or.kr/bd/ab/bdabf001cv.do (accessed on 15 June 2023) and https://data.seoul.go.kr/dataList/OA-2220/S/1/datasetView.do (accessed on 15 June 2023), respectively.
